# Peripheral and tumor‐infiltrating immune cells are correlated with patient outcomes in ovarian cancer

**DOI:** 10.1002/cam4.5590

**Published:** 2023-01-16

**Authors:** Weiwei Zhang, Yawen Ling, Zhidong Li, Xingchen Peng, Yazhou Ren

**Affiliations:** ^1^ Department of Biotherapy and National Clinical Research Center for Geriatrics, Cancer Center West China Hospital, Sichuan University Chengdu China; ^2^ Department of Oncology, Cancer Prevention and Treatment Institute of Chengdu Chengdu Fifth People's Hospital (The Second Clinical Medical College, Affiliated Fifth People's Hospital of Chengdu University of Traditional Chinese Medicine) Chengdu China; ^3^ School of Computer Science and Engineering, Shenzhen Institute for Advanced Study University of Electronic Science and Technology of China Chengdu China

**Keywords:** machine learning, ovarian cancer, peripheral blood, tumor‐infiltrating immune cells

## Abstract

**Objective:**

At present, there is still a lack of reliable biomarkers for ovarian cancer (OC) to guide prognosis prediction and accurately evaluate the dominant population of immunotherapy. In recent years, the relationship between peripheral blood markers and tumor‐infiltrating immune cells (TICs) with cancer has attracted much attention. However, the relationship between the survival of OC patients and intratumoral‐ or extratumoral‐associated immune cells remains controversial.

**Methods:**

In this study, four machine‐learning algorithms were used to predict overall survival in OC patients based on peripheral blood indicators. To further screen out immune‐related gene and molecular targets, we systematically explored the correlation between TICs and OC patient survival based on The Cancer Genome Atlas database. Using the TICs score method, patients were divided into a low immune infiltrating cell group and a high immune infiltrating cell group.

**Results:**

The results showed that there was a significant statistical significance between the peripheral blood indicators and the survival prognosis of OC patients. Survival analysis showed that TICs play a crucial role in the survival of OC patients. Four core genes, CXCL9, CD79A, MS4A1, and MZB1, were identified by cross‐PPI and COX regression analysis. Further analysis found that these genes were significantly associated with both TICs and survival in OC patients.

**Conclusions:**

These results suggest that both peripheral blood markers and TICs can be used as prognostic predictors in patients with OC, and CXCL9, CD79A, MS4A1, and MZB1 may be potential therapeutic targets for OC immunotherapy.

## Graphical abstract

This study combines several machine‐learning and bioinformatic analysis methods to explore the correlation of peripheral and tumor‐infiltrating immune cells with patient outcomes in ovarian cancer. The results suggest that both peripheral blood markers and TICs can be used as prognostic predictors in patients with ovarian cancer, and CXCL9, CD79A, MS4A1, and MZB1 may be potential therapeutic targets for ovarian cancer immunotherapy.

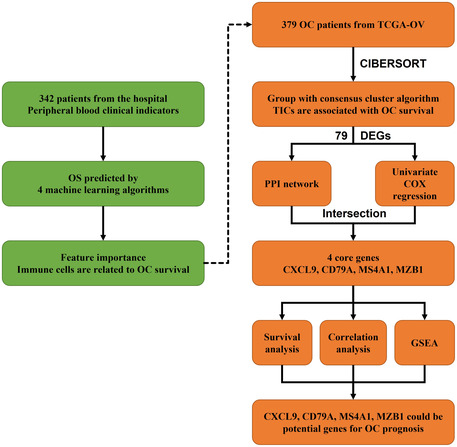

## 1 INTRODUCTION

Ovarian cancer (OC), one of the most lethal gynecologic malignancies in the world has been increasing in incidence and mortality.^1^ In 2020, there were approximately 313,959 new cases of OC diagnosed and 207,252 deaths worldwide.^2^ Due to the ovaries being located in the pelvis, early symptoms are not obvious. Combined with the lack of effective early cancer screening techniques, more than 70% of patients are already at an advanced stage at the time of diagnosis.^3^ High recurrence rates, high mortality, and limited treatment options are the main reasons for the short survival time of OC patients, and they are also huge challenges for clinicians. Therefore, there is an urgent need to find relevant biomarkers to better predict the survival prognosis of OC.

Although surgery and chemotherapy are initially effective treatments for most OC patients, 70%–80% of patients will succumb to recurrence with chemotherapeutic resistance within 2–5 years after the initial treatment, due to the heterogeneity of OC.^4^, ^5^ In recent years, immunotherapy alone or in combination with other regimes for various cancers is quickly evolving, such as head and neck squamous cell carcinoma,^6^ gastric,^7^ kidney cancer,^8^ liver cancer,^9^, ^10^ and non‐small‐cell lung cancer (NSCLC).^11^, ^12^, ^13^, ^14^ Although promising therapies such as immune checkpoint inhibitors (ICIs), chimeric antigen receptors (CARs), and TCR‐engineered T cells have achieved excellent results in lymphomas or other solid tumors, the immune treatment response rates remain moderate in OC patients. Nonetheless, OC has been considered an immunogenic disease, nonspontaneous antitumor immune responses could be found in the tumor microenvironment or peripheral blood.^15^, ^16^, ^17^, ^18^ Immune cells in peripheral blood, tumor microenvironment, or ascites play a key role in ovarian carcinogenesis, including lymphocytes, regulatory T cells, natural killer (NK) cells, and tumor‐associated macrophages.^19^ The lack of effective prognostic indicators and screening indicators for immunotherapy‐advantaged populations may be one of the reasons for the poor benefit of OC immunotherapy.

So far, the only predictive biomarker to judge the efficacy of immunotherapy is the expression of programmed cell death ligand 1 (PD‐L1) assessed by IHC from tissue sections. Some investigators have also proposed tumor mutational burden (TMB) detected from tumor tissue as another potential novel biomarker.^20^, ^21^, ^22^ Nonetheless, there are many limitations to the use of PD‐L1 and TMB. First, technology and tumor biology reasons are a challenge for pathologists.^23^, ^24^ The acquisition of tumor specimens is invasive and difficult, insufficient tissue specimens or low tumor cell content may affect the detection results of PD‐L1 and TMB. Secondly, PD‐L1 is expressed in both tumor and immune cells, and its expression varies intra‐ and intertumor, complicating the evaluation of the efficacy of immunotherapy.^25^, ^26^ Third, PD‐L1 expression in small biopsy tumors was not representative of the entire tumor.^25^ Therefore, some patients may not receive immunotherapy due to tissue sampling bias. Furthermore, although some patients have high TMB and PD‐L1 expression, immunotherapy may still fail, which is related to the complexity of tumor immunopathology.^27^ Therefore, it is an urgent need to identify specific prognostic biomarkers and potential modifiers in OC patients.

With the development of technology, liquid biopsy (LB) has been developed to predict biomarkers in various cancers, especially lung cancer.^28^ LB is a very attractive method to detect circulating tumor DNA (ctDNA), circulating tumor cells, methylation markers, T cells, proteins, exosomes, or other gene molecules in the blood to guide tumor treatment decisions, early detection of tumors, monitoring of tumor recurrence and evaluation of efficacy.^29^ Although LB has great significance and broad prospects in the individualized management of tumor patients, ctDNA and minimal residual disease (MRD) detection are expensive, technically difficult, and relatively poorly ubiquitous. However, a recent study found a quick way to obtain neoantigen‐specific T cells by isolating T cells from the peripheral blood of patients.^30^ Such neoantigen‐specific T cells can not only treat cancer but also lay the foundation for the strategy of autologous cell immunotherapy in the cancer field by combining LB with tumor immunotherapy. Therefore, we believe that the combination of peripheral blood markers and immune‐related gene molecules is a low cost and reproducible LB method.

Nowadays, several studies have shown that peripheral blood cells such as lymphocytes and tumor‐infiltrating immune cells (TICs) are closely related to the efficacy of immunotherapy and can be predictors of overall survival in cancer patients. Wang's study demonstrated that the combination of serum platelet/lymphocyte ratio (PLR), carbohydrate antigen 125 (CA125), and diffusion‐weighted imaging (DWI) can provide a strong reference for the diagnosis of OC recurrence.^31^ Additionally, Kim's research has developed web‐based nomograms of peripheral blood markers such as platelets (PLT), neutrophils (NEU), lymphocytes (LC), and monocytes to predict treatment response and prognosis in OC.^32^ Many studies have shown that TICs, including T cells, macrophages, dendritic cells, neutrophils, and NK cells, play a key role in the prognosis of OC patients.^33^, ^34^ Immune cells, cytokines, and genetic information in peripheral blood are very rich, and their changes can fully reflect the body's response to malignant tumors before and after treatment.

Therefore, this study employed four machine‐learning algorithms to explore the correlation between peripheral blood indicators and the survival of OC patients. Next, using the RNA‐Seq data in the Cancer Genome Atlas (TCGA) database, the correlation between TICs and the prognosis of OC patients was further analyzed by the method of TICs score. Four core genes related to immunity were finally identified. These peripheral blood immune cells and immune‐related genes may provide some guidance for OC immunotherapy strategies, and may also become new prognostic markers.

## 2 MATERIALS AND METHODS

### 2.1 Raw data

Preoperative peripheral blood clinical indicators data in this study came from 342 patients with OC who were diagnosed and treated by the Fifth People's Hospital of Chengdu from 2005 to 2015. The ethics committee of the Fifth People's Hospital of Chengdu approved the study protocol. All patients were treated with cytoreductive surgery and platinum‐based chemotherapy. Excluded patients included acute infections, co‐morbidities, autoimmune diseases, and hematologic diseases. Platinum resistance was defined as the patient who relapsed within 6 months after the end of first‐line platinum‐based chemotherapy, whereas all other patients were defined as platinum‐sensitive. The OS was defined as the time from treatment to the date of death or last follow‐up. All OC patients were followed up every 3 months for the first 2 years and every 6 months thereafter until June 2017.

The average age of the patients is 51.93 years (24–76), and the average overall survival time is 43.2 months. Among these patients, there are 35 cases (10.2%) in stage I, 52 cases (15.3%) in stage II, 208 cases (60.8%) in stage III, and 47 cases (13.7%) in stage IV. The clinical indicators of the patients include age, cancer stage, chemoresistance, CA‐125 serum concentration level (CA‐125), surgical satisfaction (diameter of the largest residual mass <2 cm; diameter of the largest residual mass ≥2 cm), ascites, histological grade, histological type (serous; other types, such as mucus, clear cell, etc.), C‐reactive protein concentration (CRP), platelet (PLT) count, lymphocyte (LC) count, neutrophil (NEU) count, albumin (ALB) count, hemoglobin (HGB) content, basophil (BASO) count, monocyte (Mono) count, and white blood cell (WBC) count. PNI was calculated as the serum albumin (g/L) + 0.005 × lymphocyte count (per mm) in the peripheral blood. LMR was defined as the absolute lymphocyte count/absolute monocyte ratio. PLR was defined as the absolute platelet count to absolute lymphocyte count ratio. NLR was defined as the absolute neutrophil count to absolute lymphocyte count ratio. Body mass index (BMI) was defined as weight/height^2^. All the clinical indicators of the OC patients are listed in Table 1.

**TABLE 1:** Clinical indicators of the OC patients

Clinical indicators	Values/distribution
Age (years)	Mean: 51.93 (24–76)
Status	
Dead	249
Alive	93
Stage	
I	35
II	52
III	208
IV	47
Chemosensitivity	
Sensitive	204
Resistant	35
Missing	3
CA‐125 (U/ml)	71
<35	271
≥35	
Surgical satisfaction	
Diameter of largest residual mass <2 cm	189
Diameter of largest residual mass ≥2 cm	153
Ascites	
No	141
Yes	201
Histological grade	
G1	122
G2	78
G3	142
Histological subtype	
Serous	204
Others	138
CRP (mg/L)	Mean: 34.26 (0–251.7)
PLT (×10^9^/L)	Mean: 334.10 (102–710)
LC (×10^9^/L)	Mean: 1.67 (0.51–4.77)
NEU (×10^9^/L)	Mean: 5.32 (1.66–16.9)
ALB (g/L)	Mean: 37.33 (22–52)
HGB (g/L)	Mean: 115.00 (21.6–150)
BASO (×10^9^/L)	Mean: 0.04 (0–1.41)
Mono (×10^9^/L)	Mean: 0.55 (0.07–6.01)
WBC (×10^9^/L)	Mean: 7.28 (0.9–18.9)
BMI	Mean: 22.22 (14.95–37.72)
PLR	Mean: 233.11 (45.74–913.85)
NLR	Mean: 3.71 (0.78–15.50)
PNI	Mean: 45.62 (26.60–65.60)
LMR	Mean: 4.24 (0.20–28.33)

Abbreviations: ALB, albumin; BASO, basophil; BMI, body mass index; CRP, C‐reactive protein concentration; HGB, hemoglobin; LC, lymphocyte; LMR, lymphocyte to monocyte ratio; Mono, monocyte; NEU, neutrophil; NLR, neutrophil to lymphocyte ratio; PLR, platelet to lymphoid count ratio; PLT, platelet count; PNI, prognostic nutritional index; WBC, white blood cell.

Transcriptome RNA‐seq data (HTSeq‐FPKM) and its related clinical data of 379 OC patients in TCGA‐OV were downloaded from the TCGA database (https://portal.gdc.cancer.gov/).

### 2.2 Evaluation of TICs

CIBERSORT^35^ algorithm was employed to evaluate the proportion of 22 TICs in OC cases, and cases with *p* value <0.05, which indicated that the deconvolution results were accurate, were selected for further analysis.

### 2.3 Consensus clustering for grouping

The consensus cluster algorithm was adopted by using ConsensusClusterPlus^36^ R‐package to calculate the number of clusters in the tumor samples based on the proportion of TICs and grouped to further explore the correlation between the immune microenvironment and OC.

### 2.4 Survival analysis

Survival analysis was performed using survival and survminer R‐package. Survival curves were plotted using the Kaplan–Meier method and *p* value <0.05 was considered significant.

### 2.5 Generation of DEGs between different groups

Performed differential analysis by limma R‐package and compared different immune groupings to generate DEGs. DEGs with *|*logFC*|* > 1 and FDR < 0.05 were considered significant.

### 2.6 GO and KEGG enrichment analysis

With the ClusterProfiler^37^ R‐package, all DEGs were used for GO and KEGG enrichment analyses to reveal potential biological significance. Only terms satisfying both *p* value <0.05 and FDR < 0.05 were considered significantly enriched.

### 2.7 PPI network construction and univariate COX regression analysis

All DEGs were inputted into the STRING database (https://string‐db.org/) to construct protein–protein interactions (PPIs) network and then processed by Cytoscape software. With the aid of the survival R package, univariate analysis was used to analyze the prognostic impact of DEGs on patients with OC.

### 2.8 GSEA analysis

“c7.all.v7.5.symbols.gmt” was selected as the gene sets database for GSEA^38^ analysis in the software GSEA V4.2.1. Enrichment pathways with *p* value <0.05 and FDR < 0.05 were considered significant.

### 2.9 Machine‐learning algorithms

All experiments are implemented through the Python programming language (Python Software Foundation, https://www.python.org/), package LightGBM (https://lightgbm.readthedocs.io/), package XGBoost (https://xgboost.readthedocs.io/), and package scikit‐learn (https://scikit‐learn.org/stable/).

Support vector machine (SVM) is a machine‐learning model that finds the best separation hyperplane in the feature space and maximizes the interval between positive and negative samples on the training set. It is suitable for supervised classification problems.^39^

Random forest (RF) is an ensemble learning classifier based on the decision tree. It contains a specified number of decision tree classifiers, and the output category is decided by the output results of these decision trees.^40^

XGBoost is one of the boosting algorithms. The idea of boosting algorithm is to integrate a number of weak classifiers to form a strong classifier. XGBoost is a boosted tree model, which integrates many tree models to form a strong classifier, and the tree model used is the CART regression tree model.^41^

LightGBM is a fast, distributed, high‐performance gradient‐boosting framework based on a decision tree algorithm, which can be used for sorting, classification, regression, and many other machine‐learning tasks.^42^

All missing values were removed so that each algorithm can compete fairly, and OS was selected as the binary classification label. Eighty percent of the data was used for training the models and 20% was used as the test set. The hyperparameters of all models were obtained using the grid search algorithm with threefold cross‐validation based on the default parameters to obtain the optimal parameters.

## 3 RESULTS

### 3.1 The overall structure of the study

**FIGURE 1:**
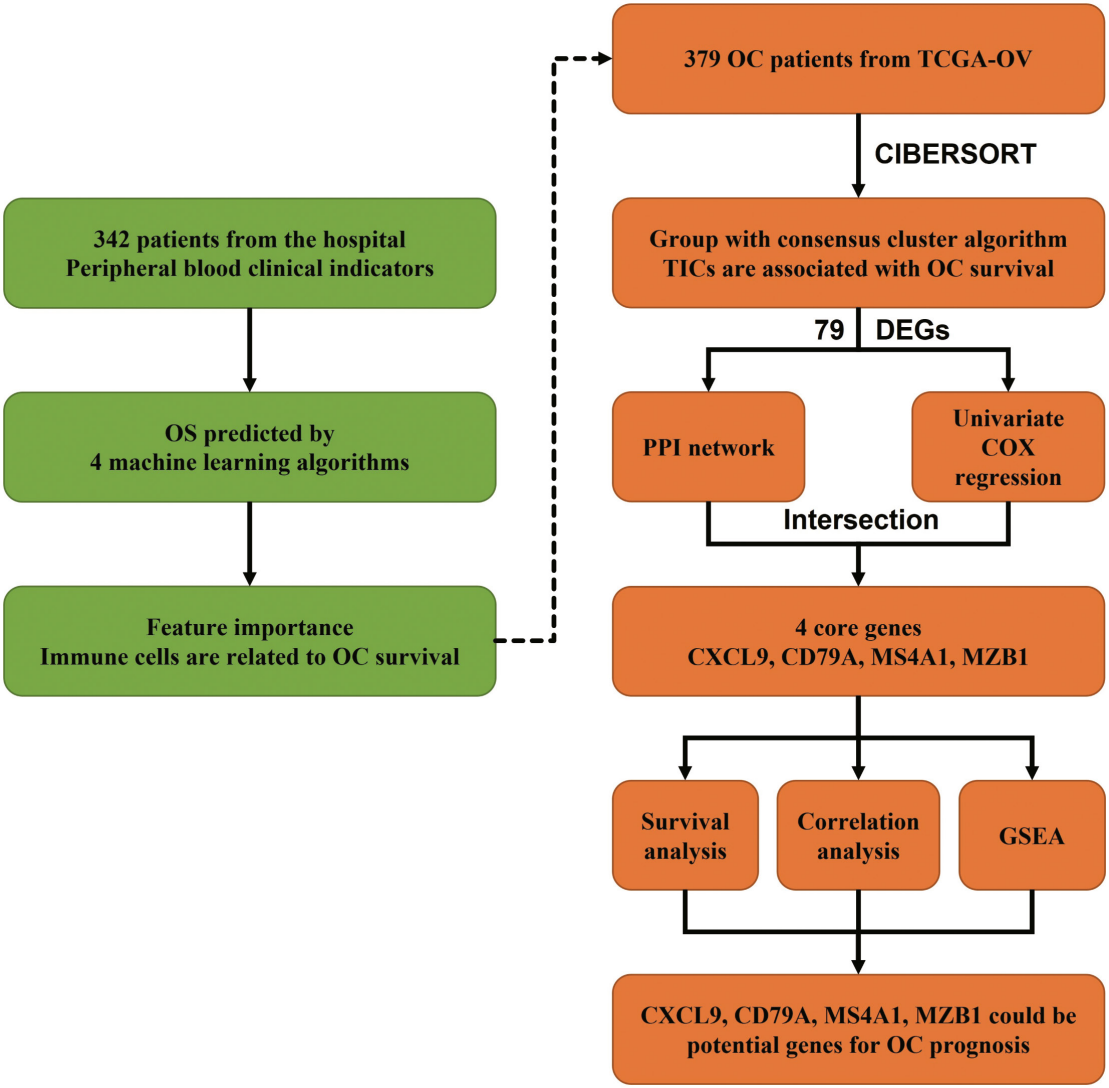
The overall structure of the study

The overall structure of the study is shown in Figure 1. First, 342 OC patients were collected from the Fifth People's Hospital of Chengdu. Four machine‐learning algorithms were applied to predict the overall survival status of patients and to give the correlation between the peripheral blood indicators and OC survival. To further explore the relationship between the immune microenvironment and the progression of OC, gene expression profiles of OC patients were subsequently downloaded from the TCGA database and then the proportion of 22 TICs in patients were evaluated based on the CIBERSORT algorithm. Then the correlation between TICs and OC survival was analyzed by Kaplan–Meier survival analysis. Combining PPI and univariate COX regression analysis based on the gene difference analysis, four core genes related to immune activity were identified. Finally, survival analysis, correlation analysis, and GSEA were performed to confirm the correlation of these genes with survival as well as immune activity of OC patients.

### 3.2 Peripheral blood cells are associated with survival of OC and their indicators can be prognostic factors

In order to explore the correlation between peripheral blood clinical indicators and overall survival (OS) of OC patients, the present study applied four common machine‐learning algorithms, which are SVM, RF, XGBoost, and LightGBM, to predict the survival of OC patients and give the feature importance based on their clinical indicators.

**FIGURE 2:**
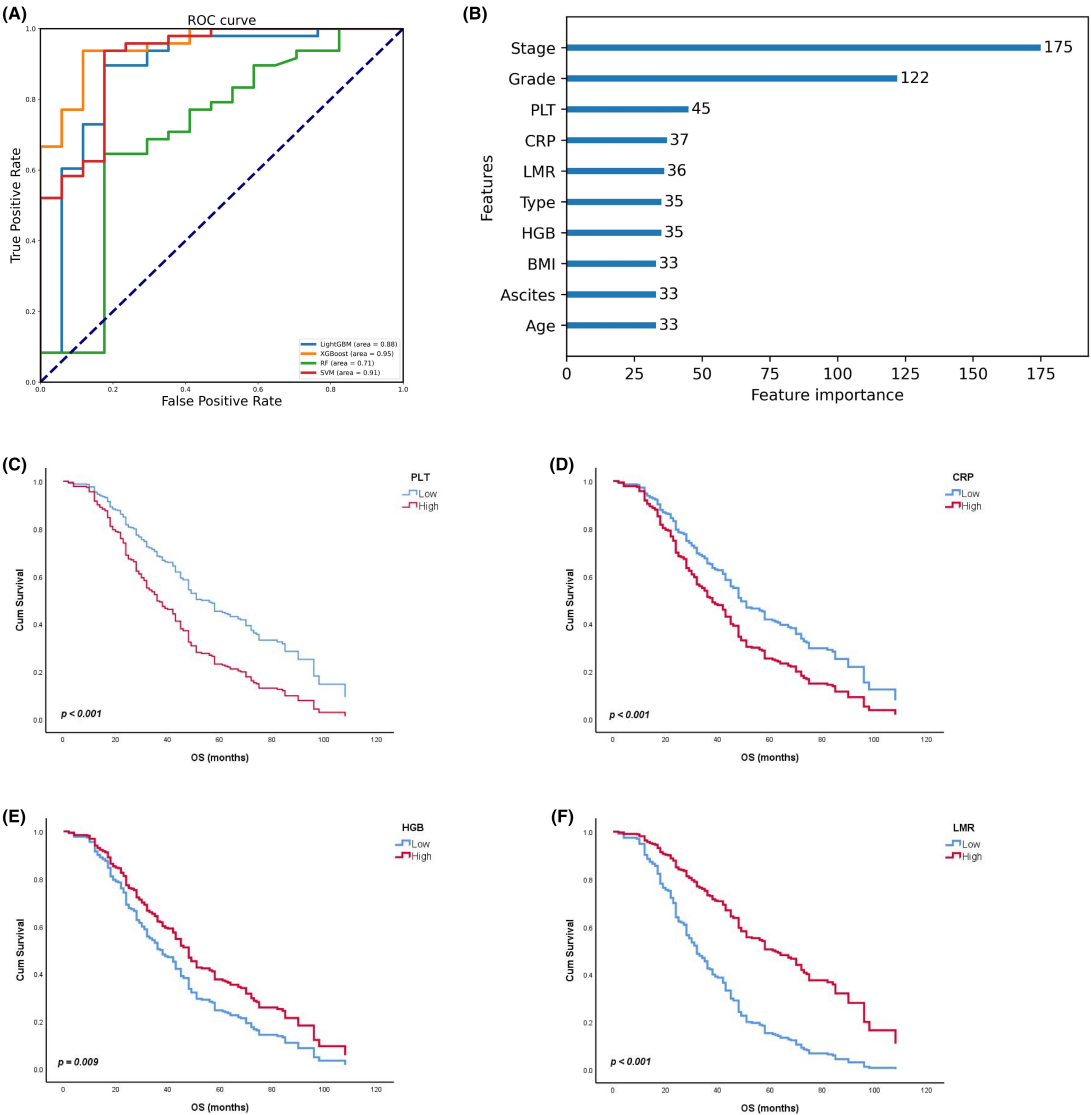
Results from machine‐learning and univariate COX analysis conducted on peripheral blood indicators. (A) ROC curve of different machine‐learning models. (B) Feature importance with LightGBM. (C) Univariate COX analysis of PLT with OS (*p* value <0.001). (D) Univariate COX analysis of CRP with OS (*p* value <0.001). (E) Univariate COX analysis of LMR with OS (*p* value <0.001). (F) Univariate COX analysis of HGB with OS (*p* value = 0.009).

The four machine‐learning algorithms are compared in Table 2 and Figure 2A. Overall, LightGBM has an overwhelming advantage in that it can accurately predict patients' OS based on the clinical indicators of OC patients. Specifically, LightGBM, XGBoost, and SVM all have high precision, and it represents the rate at which the models correctly predict a sample as dead. In addition, the samples that actually died can be correctly judged by the models with a high recall. Notably, LigthtGBM has a higher specificity, implying that the model is able to predict the surviving samples correctly, whereas the other models, especially the RF, are mediocre. F1‐score is the summed average of the precision and recall, LightGBM, XGBoost, and SVM all obtain satisfying performance results. Intuitively, it is the potential to apply machine‐learning models with the prediction of the prognosis of OC patients.

**TABLE 2:** Performance evaluation results of different models

Models	Precision	Recall	Specificity	F1‐score	Accuracy
LightGBM	**0.90**	0.94	**0.71**	**0.92**	**0.88**
XGBoost	0.87	0.96	0.59	0.91	0.86
RF	0.74	**1.00**	0.00	0.85	0.74
SVM	0.85	0.98	0.53	0.91	0.86

*Note*: The best results in each column are highlighted in boldface.

What is more, LingtGBM gave the feature importance of the top 10 clinical indicators (Figure 2B). The results showed that the clinical indicators including tumor stage, histological grade, PLT, CRP, LMR, histological type, HGB, BMI, Ascites, and Age play a more important role in the survival prediction of OC patients. As clinically evident, Stage and Grade have an important association with the OS of OC patients. Except for the Type, BMI, Ascites, and Age, the rest features, including PLT, CRP, LMR, and HGB, are all peripheral blood‐related indicators, implying that peripheral blood clinical indicators may be associated with the prognosis of OC patients. In order to validate the obtained results and conclusions, univariate COX analysis of peripheral blood immune cell indicators with OS was implemented on software SPSS 26.0. As shown in Figure 2C–F, clinical peripheral indicators, including PLT, CRP, LMR, and HGB, as pointed out by LightGBM, were significantly associated with the survival of OC patients (*p* value <0.05). Altogether, the survival of OC patients is influenced by peripheral blood indicators, and the manifestation of these blood cells in the peripheral can provide a basis for the prognosis of OC.

### 3.3 Immune microenvironment are correlated with the survival of OC patients

**FIGURE 3:**
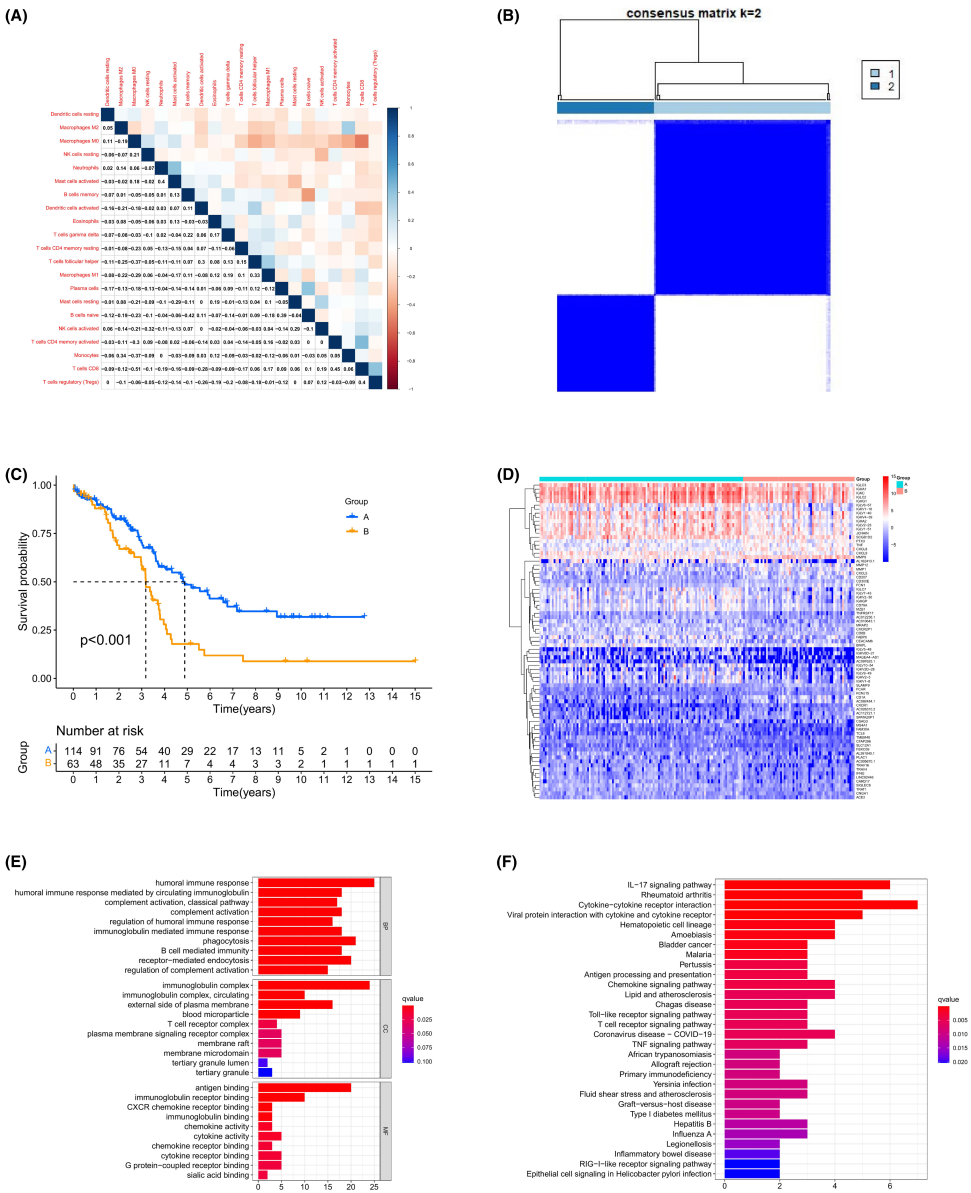
Immune microenvironment is correlated with the survival of OC patients. (A) Heat map of 22 TICs correlation. Values in the boxes indicate the correlation *p* value between two TICs, and colors indicate the degree of correlation. (B) Clustering OC patients into two groups based on TICs profile. (C) Correlation of immune microenvironment with the survival of OC patients (*p* value <0.001). (D) Heatmap of the DEGs. Each column represents a sample, each row represents the expression of a gene. Genes with high expression are colored red, and genes with low expression are colored blue. (E) GO enrichment analysis. (F) KEGG enrichment analysis.

For the OC patients downloaded from the TCGA database, the CIBERSORT algorithm was first adopted to analyze the immune microenvironment. By analyzing the proportion of tumor‐infiltrating immune subsets, 22 immune cells' expression profiles in OC patients, and a correlation heat map can be constructed (Figure S1, Figure 3A). Subsequently, 177 samples were divided into two groups (114 samples in group A and 63 samples in group B) based on the proportion of TICs in OC patients with consensus clustering, which is a common unsupervised clustering method (Figure 3B). To explore the correlation between the immune microenvironment and the survival of patients with OC, Kaplan–Meier survival analysis was applied to the grouping of TICs. As shown in Figure 3C, the immune microenvironment was significantly associated with the survival of patients with OC (*p*‐value <0.001), and patients in group A had a higher survival rate while those in group B had a lower survival rate. This implies that TICs in the immune microenvironment can indicate the prognosis of OC patients.

### 3.4 Enrichment of DEGs implies that survival of OC patients is influenced by TICs and related pathways

To determine the differences in the expression of different genes between immune microenvironment groups, a differential analysis was implemented. A total of 79 DEGs were obtained, consisting of 19 upregulated genes and 60 downregulated genes (Figure 3D). For the obtained DEGs, enrichment analyses were performed to see the main functional pathways of these genes. The results of gene ontology (GO) enrichment analysis (Figure 3E) showed that these DEGs were mainly enriched in humoral immune response, immunoglobulin complex and antigen binding, etc., which are all immune‐related. In addition, results from the Kyoto Encyclopedia of Genes and Genomes (KEGG) (Figure 3F) suggested that cytokine–cytokine receptor interaction, chemokine signaling pathway, IL‐17 signaling pathway, etc. are the main enrichment pathways. Taken together, the enrichment results showed that 79 DEGs were mainly associated with immune activity, suggesting that the survival of OC patients is influenced by the expression of TICs and related pathways.

### 3.5 Intersection analysis of PPI network and univariate COX regression

**FIGURE 4:**
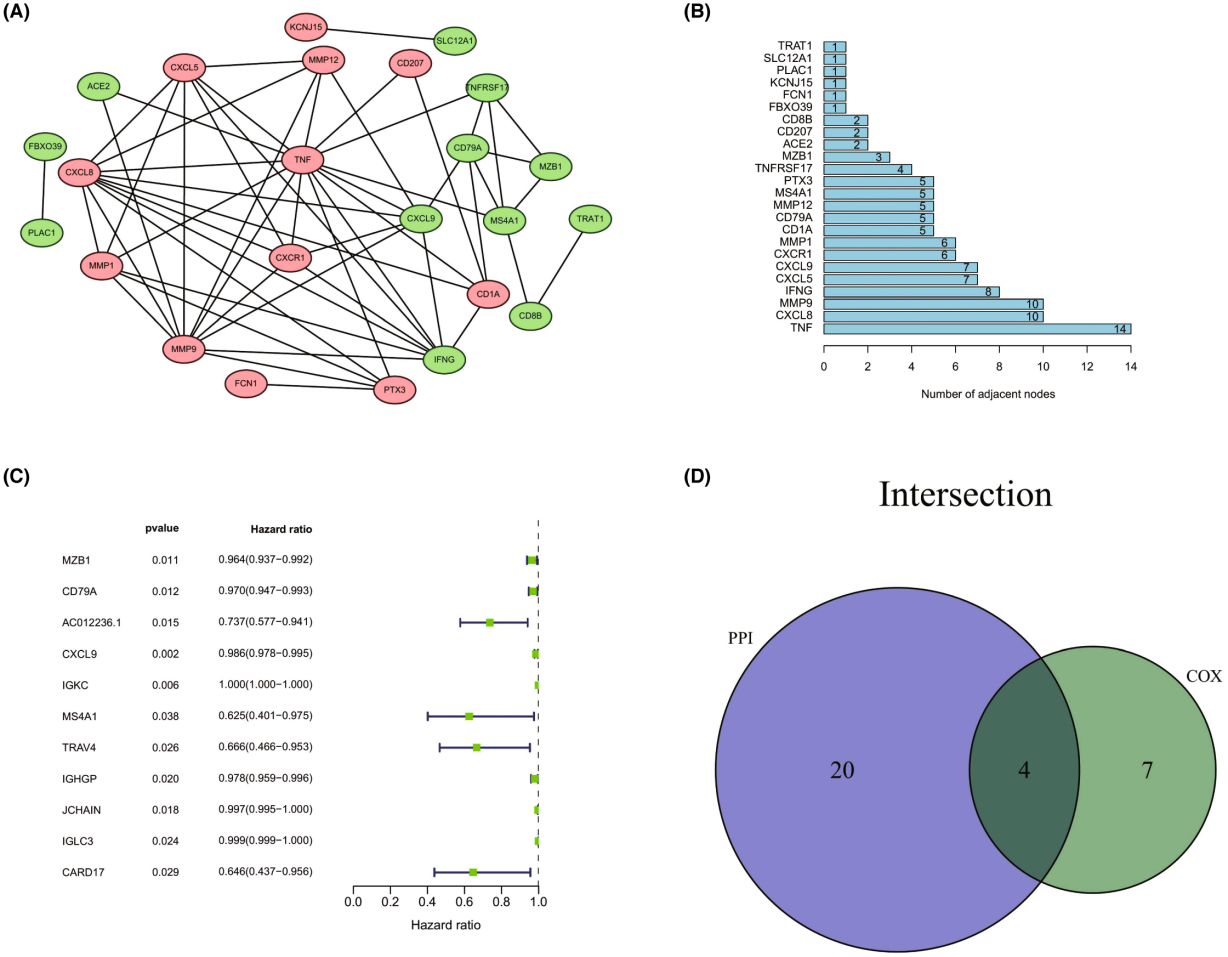
Intersection analysis of PPI network and univariate COX regression. (A) Protein–protein interaction (PPI) network of DEGs. (B) Count of adjacent nodes. (C) Univariate COX regression analysis of 79 DEGs (*p*‐value <0.05). (D) Venn plot of the nodes in the PPI network and the significant factors in the univariate COX analysis.

A PPI network of DEGs with medium confidence (0.400) was constructed by the online STRING database and then processed by Cytoscape software. The obtained PPI network consists of 24 nodes (12 upregulated genes and 12 downregulated genes) and 56 edges (Figure 4A). Further, the number of adjacent nodes of each node in the PPI network was counted, and the results are presented in Figure 4B. The higher the count of nodes implies the more important their regulatory role in the network. In addition, univariate COX regression was performed to analyze the survival of OC patients with the obtained 79 DEGs to identify significant factors associated with prognosis (Figure 4C). Subsequently, the nodes in the PPI network were intersected with the significant factors in the univariate COX analysis, and a total of four common genes were obtained, that is, CXCL9, CD79A, MS4A1, and MZB1 (Figure 4D).

### 3.6 The four core genes significantly associated with survival and TICs in OC patients

**FIGURE 5:**
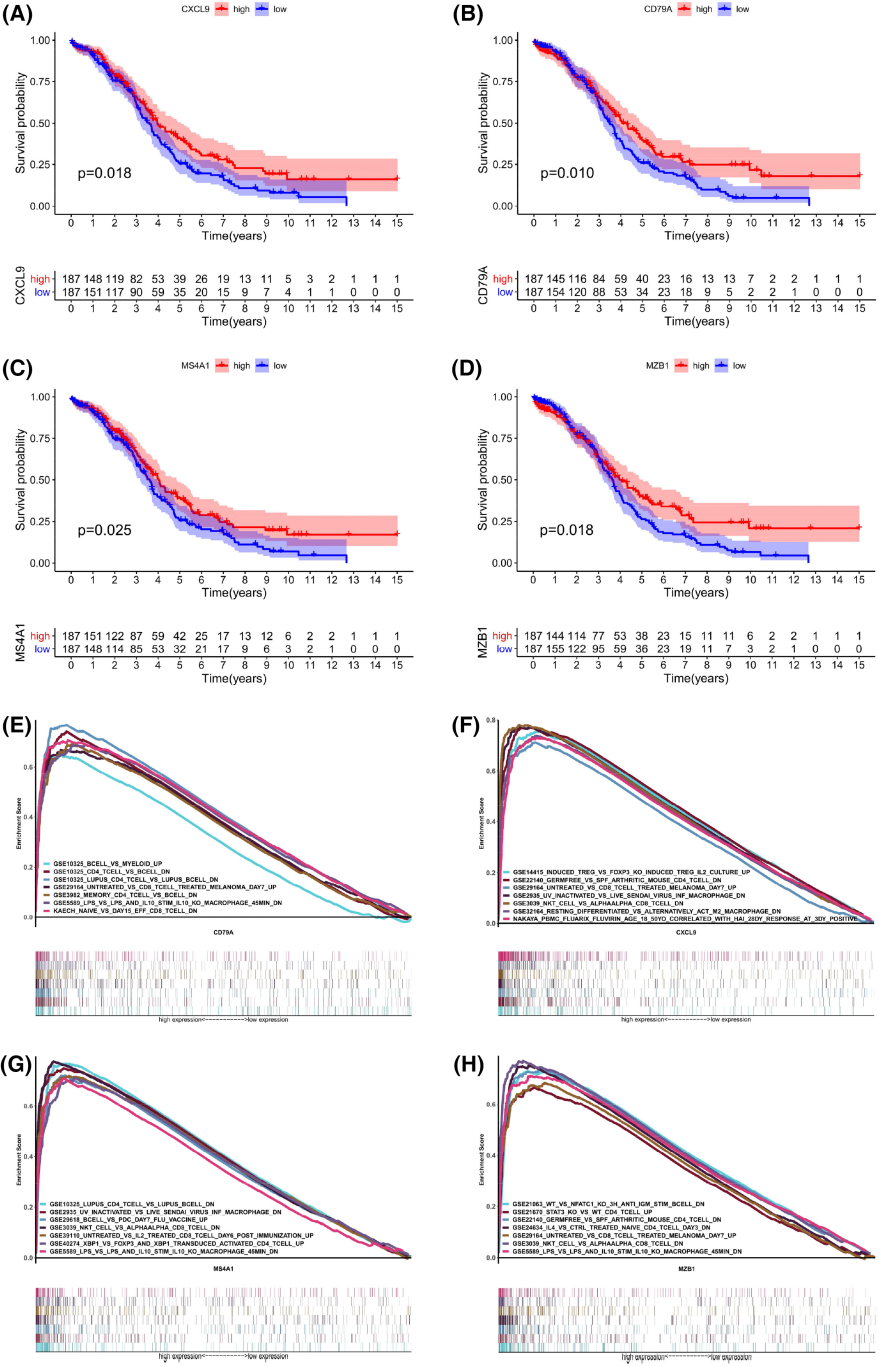
Correlation analysis and gene set enrichment analysis (GSEA) of the four core genes. (A) Correlation of the CXCL9 with the OC survival (*p* value = 0.018), (B) correlation of the CD79A with the OC survival (*p* value = 0.010), (C) correlation of the MS4A1 with the OC survival (*p* value = 0.025), (D) correlation of the MZB1 with the OC survival (*p* value = 0.018), (E) GSEA of the CXCL9, (F) GSEA of the CD79A, (G) GSEA of the MS4A1, (H) GSEA of the MZB1. Only pathways with *p* value and FDR < 0.05 are considered to be enriched significantly.

**FIGURE 6:**
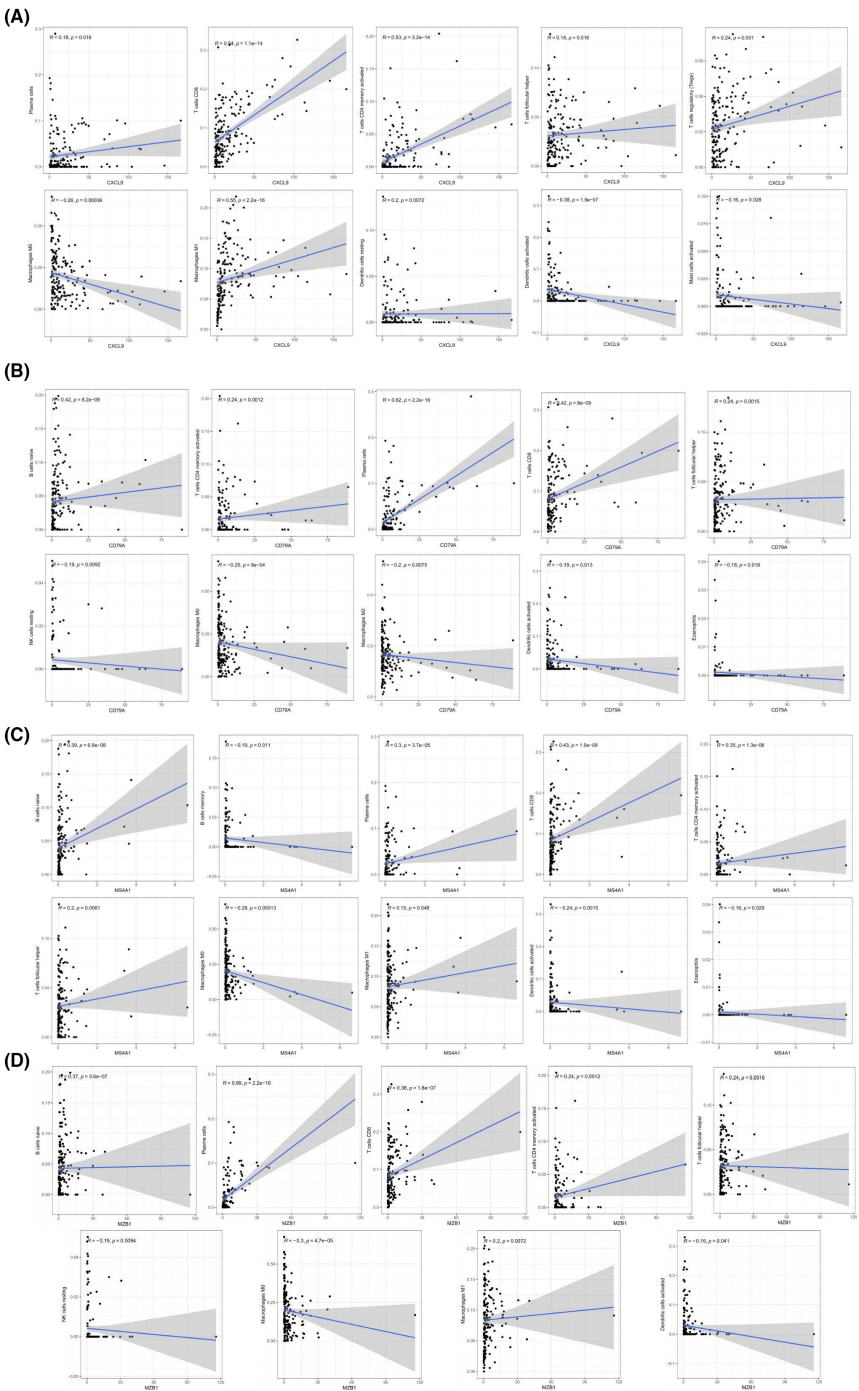
Correlation analysis of the four core genes with TICs. (A) Correlation analysis of CXCL9 with TICs, (B) correlation analysis of CD79A with TICs, (C) correlation analysis of MS4A1 with TICs, (D) correlation analysis of MZB1 with TICs. The scatter plot shows the correlation of different TICs proportion with the gene expression. The blue line is the linear fitting of TICs proportion with gene expression, and the correlation with *p* value <0.05 is retained.

To explore the prognostic correlation of the obtained four core genes to OC patients, this study divided the gene expression into high‐ and low‐expression groups compared with the median expression and then implemented survival analysis. As shown in Figure 5A,B, all four core genes were significantly associated with the survival of OC patients, implying that the expression of these genes influenced the survival of OC patients. Further, four core genes were analyzed for correlation with the immune microenvironment. The results in Figure 6A and Table S1 showed that CXCL9 was correlated with plasma cells, T cells CD8, T cells CD4 memory activated, T cells follicular helper, T cells regulatory (Tregs), macrophages M0, macrophages M1, dendritic cells resting, dendritic cells activated, and mast cells activated. CD79A was correlated with B cells naïve, plasma cells, T cells CD8, T cells CD4 memory activated, T cells follicular helper, NK cells resting, macrophages M0, macrophages M2, dendritic cells activated and eosinophils (Figure 6B, Table S2). MS4A1 was correlated with B cells naïve, B cells memory, plasma cells, T cells CD8, T cells CD4 memory activated, T cells follicular helper, Tregs, macrophages M0, macrophages M1, dendritic cells activated and eosinophils (Figure 6C, Table S3), and MZB1 was correlated with B cells naive, plasma cells, T cells CD8, T cells CD4 memory activated, T cells follicular helper, NK cells resting, macrophages M0, macrophages M1, and dendritic cells activated (Figure 6D, Table S4). All four genes were significantly associated with T cell and macrophage cell or B cell‐related functions. Specifically, they were positively correlated with, for example, T cells CD8, and macrophages M1 and negatively correlated with macrophages M0.

### 3.7 Identify signaling pathways of the four genes involved in

To identify the signaling pathways in which these four core genes, that is, CXCL9, CD79A, MS4A1, and MZB1, may be involved, gene set enrichment analysis (GSEA)^43^ was implemented in high and low expression groups according to the gene expression median. GSEA analysis illustrated that all four genes were significantly associated with immune‐related signaling pathways (Figure 5E–H). CXCL9, MS4A1, and MZB1 were all enriched in NKT_CELL_VS_ALPHAALPHA_CD8_TCELL_DN. Taken together, all four core genes were associated with B cell, T cell, and macrophage corresponding pathways. These enrichment results implied that CD79A, CXCL9, MS4A1, and MZB1 were not only associated with TICs but also involved in important immune pathways.

## 4 DISCUSSION

The diagnosis and treatment of OC remain major challenges for clinicians.^2^, ^44^ Despite the increasing number of treatment options for OC, such as immunotherapy, molecular‐targeted therapy, and poly‐ADP‐ribose polymerase (PARP) inhibitors, the survival rate of OC patients has not improved significantly.^45^ This may be due to the lack of recognized effective tumor biomarkers, especially early screening indicators and real time‐monitoring prognostic recurrence indicators. Currently, FDA‐approved predictive biomarkers for OC only include CA‐125 and HE4.^46^ With the success of immune checkpoint blockade and CAR‐T cell therapy in many cancer patients, researchers have begun to study the relationship between the immune microenvironment, immune score, immune cells, chronic inflammation, and tertiary lymphoid structures and the development of tumors, thereby further improving the efficacy of immunotherapy. However, we all know that the immune system is critical to the development and progression of OC, and immune escape and resistance can be caused by immune dysregulation.^5^, ^47^ A growing number of evidence revealed that the state of the immune microenvironment is inextricably related to the prognosis of OC.^48^, ^49^ Therefore, there is a need to further understand the mechanisms of the immune microenvironment in OC and to discover better prognostic biomarkers.

In the era of immuno‐oncology, LB is undoubtedly a promising detection method, and some positive results are promoting its translation from laboratory to clinical practice.^50^ Because LB specimens are derived from blood, urine, etc., they are easy to obtain and have good repeatability. Therefore, it is more conducive to real‐time tracking and monitoring of the treatment effect on patients. LB is not limited to biomarkers such as ctDNA, micro‐RNAs (miRNA). or long noncoding (lncRNA), which have been shown to increase the chances of identifying the presence of targetable mutations and help assess treatment response or monitor relapse.^51^, ^52^ Chaudhuri's study shows that ctDNA profiling can early detect MRD in localized lung cancer.^53^ A Prospective Multicenter Cohort Study has demonstrated that perioperative ctDNA analysis is effective in the early detection of MRD and relapse risk stratification of NSCLC.^54^

However, there are still some gaps and challenges to truly integrate ctDNA or MRD into clinical practice.^55^ First, most of the current studies on ctDNA and MRD are small studies, and more multicenter, randomized, prospective studies are still needed to further explore its clinical value. In addition, the sensitivity, specificity, and economic cost are still the main reasons that limit the widespread application of ctDNA or MRD detection in the clinic. At present, it is still a major difficulty to screen out comprehensive prognostic indicators that are economical, reproducible, sensitive, and specific. Bodor's study reviews the biomarker PD‐L1 as well as other tissue‐ and serum‐based markers, such as cytotoxic T‐lymphocyte‐associated antigen 4, interferon γ, NK cells, and tumor‐infiltrating lymphocytes, that have the potential to better predict responders to immunotherapy.^56^ From the perspective of economy and popularity, the LB method combining peripheral blood indicators and prognosis‐related genes is a promising and valuable method. To explore better survival prognostic biomarkers in OC patients, we performed a comprehensive analysis of peripheral blood markers and immune‐infiltrating cells in the tumor microenvironment in this study and screened out prognosis‐related immune genes.

Contemporarily, machine learning has become a powerful helper in the medical field, showing great potential in disease diagnosis,^57^, ^58^ medical image processing,^59^, ^60^ etc. In this present study, four machine‐learning algorithms including SVM, RF, XGBoost, and LightGBM were applied to predict the OS of OC patients. The performance evaluation results demonstrated on the one hand that machine learning can make a big difference in the prediction of OC patient survival, and on the other hand that the survival of OC patients can be accurately predicted by peripheral blood clinical indicators, meaning that they are important prognostic factors. Further, with the help of the feature importance given by LightGBM, PLT, CRP, LMR, and HGB are the most relevant peripheral blood indicators for OC patient survival. Univariate COX analysis also demonstrated the significant correlation of these indicators with OC survival. Most of these are immune‐related indicators, suggesting that clinical peripheral blood immune cells play a significant role in OC progression.

Immune cells infiltrating within tumor tissue are extravasated from peripheral blood, and thus TICs are more responsive to their relationship with OC progression and patient survival. Recently, the importance of TICs in cancer progression has been revealed by many researches.^61^, ^62^, ^63^ Immune cell infiltration in tumors is strongly correlated with clinical outcome, and therefore, TICs are most likely to contribute as biomarkers to improve the prognosis of OC. In this view, this study utilized the CIBERSORT algorithm to estimate the proportion of 22 TICs in OC patients. To explore the collaborative interaction between TICs in the immune microenvironment and OC development and progression, the consensus clustering algorithm was applied to group OC patients. The goal of clustering is to make the differences within groups as small as possible and the differences between groups as large as possible. Thus, clustering groups can be better for subsequent analysis than grouping based on median values. Survival analysis reflected a dramatic difference in OC patient survival between the two groups. Patient survival was significantly higher in group A than in group B, implying a significant association between TICs and the progression of OC. What is more, GO and KEGG analysis of DEGs between the two groups demonstrated that the survival of patients was mainly influenced by immune‐related functions. The results coincide with recent studies.^48^, ^49^, ^64^, ^65^

Although only a small number of OC patients can benefit from immunotherapy, there is no doubt that immunotherapy is still the most promising and expected treatment method. CD47 has been reported to be a promising immune checkpoint and a targeted immunotherapeutic agent against OC.^66^, ^67^ It helps promote OC cell growth and motility, and its clinical relevance in OC depends on anti‐CD47 treatment.^67^, ^68^ HuNb1‐IgG4, antagonists of the CD47‐SIRP α signaling pathways, may be a potential candidate for OC clinical trials.^69^ Moreover, the discovery of receptors such as CTLA‐4,^70^ IL‐10,^71^ and NID1,^72^ among others, has provided new ideas and options for immunotherapy in OC patients. However, despite the biology of the tumor suggesting that OC patients may benefit from immunotherapy, the results of clinical trials are not satisfying and the underlying mechanisms are still not clear.^73^, ^74^

Therefore, exploring the collaborative interaction between TICs in the immune microenvironment and OC and identifying heterogeneous biomarkers to improve the prognosis of OC remains a very important and urgent issue. In the present study, four genes associated with OC immunity, that is, CXCL9, CD79A, MS4A1, and MZB1, were identified by the combined PPI and univariate COX regression analysis. The expression of the four genes was significantly associated with the survival of OC patients. Specifically, patients with high expression had higher survival and, conversely, patients with low expression showed a poorer prognosis. Several studies have also found that these genes play an important role in immune activity. It has been found that CXCL9 is a promising target for drug development, and agents that augment paracrine CXCL9 expression can show a certain antitumor activity.^75^ CD79A has also been reported to have a high degree of lineage‐specificity for B‐cell differentiation.^76^ MS4A1 expression was found to be positively correlated with the survival of colorectal cancer patients, and T cell subsets express MS4A1 in humans.^77^ Several studies concluded that some immune behaviors directed by MZB1 can prevent cancer progression, which is a promising therapeutic target.^78^, ^79^, ^80^ In this study, the expression of these four genes was strongly associated with TICs in the immune microenvironment, especially showing a linear relationship with T cells, B cells, macrophages, etc. GSEA also provided evidence for these findings, revealing that these four genes could function as potential therapeutic genes for OC prognosis.

Although this study further clarifies the relationship of peripheral blood indicators and tumor‐infiltrating immune cells with OC and also has certain guiding significance for clinical practical application, there are still some limitations. First, our results cannot be verified at present, because they are all retrospective studies, and there is a lack of multicenter, prospective clinical controlled studies. Second, the TCGA database of OC does not include peripheral blood indicators, and the clinical data in the Chengdu Fifth People's Hospital also have no TICs and genetic information, leading to a study that cannot demonstrate the correlation between peripheral blood and TICs. In addition, most clinicians do not know machine‐learning methods, so the usage of these methods has certain limitations. However, with the continuous integration of medicine and computers in the future, computer experts will visualize the machine‐learning method or develop an APP‐like software, so as to make up for this deficiency. Furthermore, in order to investigate the important guiding role of the combination of clinical blood indicators and TICs in OC survival and prognosis, this team will carry out a multi‐center, multi‐sample prospective clinical study in the near future.

## 5 CONCLUSIONS

In summary, peripheral blood cells and TICs are correlated with the prognosis of OC patients. Machine‐learning algorithms can accurately predict survival based on clinical peripheral blood indicators of OC patients and revealed that immune cell indicators in peripheral blood are closely related to patient survival. Focusing on the immune microenvironment, this study found that TICs were significantly associated with patient survival. Furthermore, four genes were identified that they were related to immune activity, namely CXCL9, CD79A, MS4A1, and MZB1. Subsequent correlation and enrichment analyses demonstrated that high expression of these genes was associated with better patient prognosis, and the four genes were enriched in immune‐related signaling pathways. Deeper studies of these genes may provide new ideas for finding better OC biomarkers or target therapy strategies in the future.

## AUTHOR CONTRIBUTIONS

**Weiwei Zhang:** Conceptualization (equal); data curation (equal); formal analysis (equal); funding acquisition (equal); writing – original draft (equal); writing – review and editing (equal). **Yawen Ling:** Formal analysis (equal); methodology (equal); software (equal); visualization (equal); writing – original draft (equal); writing – review and editing (equal). **Zhidong Li:** Methodology (equal); writing – original draft (equal). **Xingchen Peng:** Project administration (equal); writing – review and editing (equal). **Yazhou Ren:** Conceptualization (equal); funding acquisition (equal); project administration (equal); writing – review and editing (equal).

## FUNDING INFORMATION

This work was supported in part by the National Key Research and Development Program of China (2021YFE0206600), National Natural Science Foundation of China (82172842&81672386), Sichuan Science and Technology Program (2022YFSY0012, 2021ZYCD011, 2021YFSY0008, 2021CDFZ‐25, 2021CDZG‐24), Chengdu International Science and Technology Cooperation Program (2022‐GH03‐00004‐HZ), West China Nursing Discipline Development Special Fund Project (HXHL21008), Translational medicine fund of West China Hospital (CCGZH19002), the Post‐Doctor Research Project, West China Hospital, Sichuan University (2020HXBH119), the China Medical Board and clinical research incubation project of West China Hospital (Grant#22‐482&20HXFH037), the Science and technology project of the Health planning committee of Sichuan and Chengdu (2020035&2021115), Xinglin Scholars Program of Chengdu University of Traditional Chinese Medicine (YYZX2021039), Chendu Fifth People's Hospital Scientific Research Project (KYJJ2021‐05), and Chendu Fifth People's Hospital Teaching Reform Research Project (JGZX202214).

## CONFLICT OF INTEREST

The authors declare that they have no competing interests.

## Supporting information


Figure S1
Table S1
Table S2
Table S3
Table S4

## Data Availability

Anonymized data is available from the author W. Zhang (zwwei89@126.com) upon reasonable request. The TCGA‐OV dataset analyzed in this study is available in the Cancer Genome Atlas (TCGA) database (https://portal.gdc.cancer.gov/).
