# Supplementary material for: Peripheral and tumor‐infiltrating immune cells are correlated with patient outcomes in ovarian cancer
Source: Cancer Med. 2023 Jan 16;12(8):10045–61. doi: 10.1002/cam4.5590 (PMC10166954; doi:10.1002/cam4.5590)
Supplement: Supplementary file 1 — Figure S1 Table S1 Table S2 Table S3 Table S4 [file CAM4-12-10045-s001.docx]

**Supplementary**


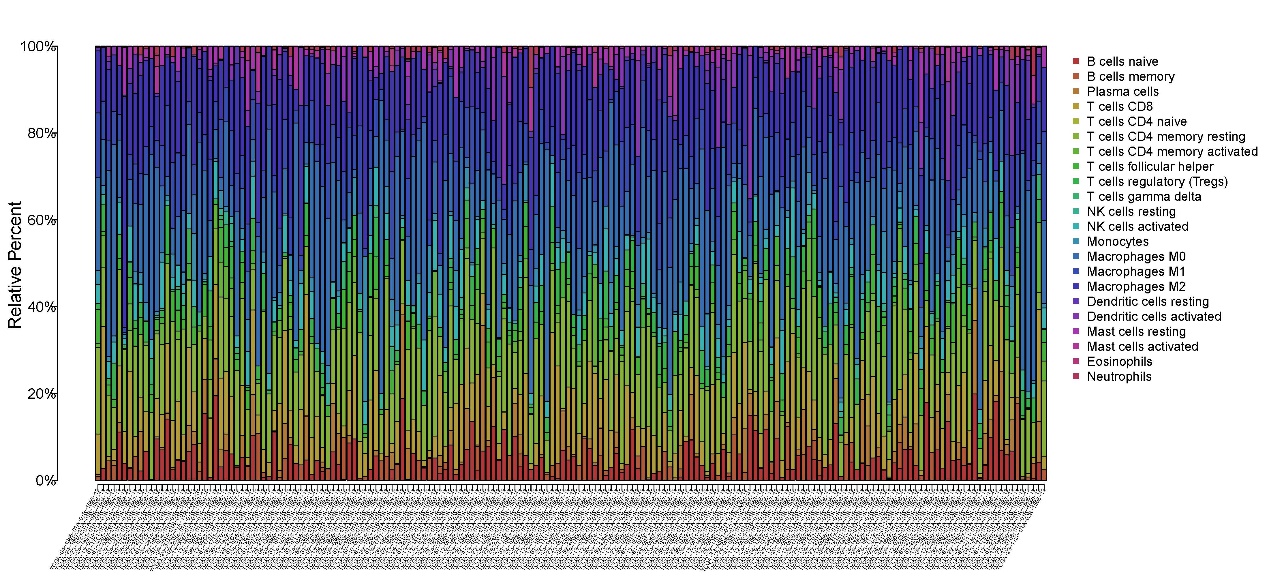


**Figure S1.** Proportion of 22 TICs in OC patients. Each column represents a sample, and the height of different colored boxes represents the corresponding TIC proportion

**Table S1.** Correlation analysis of CXCL9 with TICs.

| TICs | *p-value* |
| --- | --- |
| Plasma cells | 0.015608 |
| T cells CD8 | 1.13E-14 |
| T cells CD4 memory activated | 3.18E-14 |
| T cells follicular helper | 0.015882 |
| T cells regulatory (Tregs) | 0.000996 |
| Macrophages M0 | 0.000359 |
| Macrophages M1 | 0 |
| Dendritic cells resting | 0.007155 |
| Dendritic cells activated | 1.89E-07 |
| Mast cells activated | 0.027807 |

**Table S2.** Correlation analysis of CD79A with TICs.

| TICs | *p-value* |
| --- | --- |
| B cells naive | 8.19E-09 |
| Plasma cells | 1.74E-20 |
| T cells CD8 | 8.04E-09 |
| T cells CD4 memory activated | 0.001203 |
| T cells follicular helper | 0.0015 |
| NK cells resting | 0.009169 |
| Macrophages M0 | 0.000798 |
| Macrophages M2 | 0.007455 |
| Dendritic cells activated | 0.012891 |
| Eosinophils | 0.018379 |

**Table S3.** Correlation analysis of MS4A1 with TICs.

| TICs | *p-value* |
| --- | --- |
| B cells naive | 6.82E-08 |
| B cells memory | 0.010731 |
| Plasma cells | 3.67E-05 |
| T cells CD8 | 1.47E-09 |
| T cells CD4 memory activated | 1.28E-06 |
| T cells follicular helper | 0.00609 |
| T cells regulatory (Tregs) | 0.007453 |
| Macrophages M0 | 0.000131 |
| Macrophages M1 | 0.047596 |
| Dendritic cells activated | 0.00148 |
| Eosinophils | 0.029283 |

**Table S4.** Correlation analysis of MZB1 with TICs.

| TICs | *p-value* |
| --- | --- |
| B cells naive | 3.56E-07 |
| Plasma cells | 3.60E-26 |
| T cells CD8 | 1.76E-07 |
| T cells CD4 memory activated | 0.001196 |
| T cells follicular helper | 0.001571 |
| NK cells resting | 0.009381 |
| Macrophages M0 | 4.67E-05 |
| Macrophages M1 | 0.007244 |
| Dendritic cells activated | 0.040725 |
